# The deubiquitinase USP21 stabilizes MEK2 to promote tumor growth

**DOI:** 10.1038/s41419-018-0523-z

**Published:** 2018-04-30

**Authors:** Wenjuan Li, Kaisa Cui, Edward V Prochownik, Youjun Li

**Affiliations:** 1 Hubei Key Laboratory of Cell Homeostasis, College of Life Sciences, Wuhan University, 430072 Wuhan, China; 2 Medical Research Institute, School of Medicine, Wuhan University, 430071 Wuhan, China; 3 Division of Hematology/Oncology, Children’s Hospital of Pittsburgh of UPMC and The Department of Microbiology and Molecular Genetics, The University of Pittsburgh Medical Center, Pittsburgh, PA 15224 USA

## Abstract

Deubiquitinases (DUBs) play essential roles in normal cell proliferation and tumor growth. However, the molecular mechanisms of DUBs on hepatocellular carcinoma (HCC) remains largely unknown. In this study, based on analysis of several HCC datasets, we found that the *USP21* gene, which encodes a member of the ubiquitin-specific protease family, is highly amplified and overexpressed in HCCs, with the extent of this up-regulation significantly correlating with poor clinical outcomes. Inhibition of USP21 in HCC cell lines decreased cell proliferation, anchorage-independent growth, cell cycle progression, and in vivo tumor growth. Conversely, ectopic expression of USP21 transformed the normal human hepatocyte line HL-7702 and increased the tumorigenicity of the HCC cell line MHCC97L. Mechanistically, USP21 stabilized MEK2 by decreasing its polyubiquitination at Lys48, thereby activating the ERK signaling pathway. Importantly, MEK2 partially mediated the optimal expression of USP21-mediated oncogenic phenotypes. These findings indicate that USP21-mediated deubiquitination and stabilization of MEK2 play a critical role in HCC development.

## Introduction

Liver cancer is the fifth most common cancer worldwide and the leading cause of cancer death, with an estimated annual incidence of 782,500 new patients and 745,500 deaths^1,2^. Hepatocellular carcinoma (HCC) accounts for ~70–90% of all primary liver cancer, and it is among the most common visceral neoplasms^3,4^. Hepatitis B and C virus infections are two major risk factors for HCC, while other risk factors include aflatoxin, type 2 diabetes, alcoholic and non-alchoholic cirrhosis, fatty liver disease, and tobacco consumption^5^. Currently, surgical resection, transplantation, and percutaneous ablation are the most common treatments for patients with early-stage HCC^6^. Two important clinical features of HCC are its heterogeneity and its high rate of recurrence^7^. The survival rate of patients with HCC is very low (about 2–7%) due to a number of factors including delayed diagnosis and the development of resistant disease^2^. Therefore, improving insights into the precise molecular mechanisms of HCC pathogenesis and progression is urgent to enable early diagnosis and personalized treatment.

Protein post-translational modifications (PTMs) play important roles in controlling the activity, interactions, subcellular location and stability of many proteins. Ubiquitination is one important type of PTM that is mediated by four distinct ubiquitins and over 100 deubiquitinases (DUBs) that regulates protein functions and stability. Ubiquitination is a reversible PTM, which is critical for regulation of protein degradation, as well as cellular processes such as DNA repair, transcription, and signal transduction^8^. DUBs serve as essential controllers of various pathways involved in cancer and other diseases^8^. There are six subfamilies of DUBs in human proteome based on their sequence similarity: ubiquitin carboxyl-terminal hydrolases (UCHs), ubiquitin specific proteases (USPs), ovarian tumor-like proteases (OTUs), Josephins and JAB1/MPN/MOV34 metalloenzymes (JAMMs), and motif interacting with ubiquitin-containing novel DUB family (MINDYs)^9^. Among these DUBs, the USPs are known to be the largest subfamily^10^, with 60–70 members^11^.

USP21 belongs to the USP subfamily and plays important roles in regulation of various signaling pathways. On one hand, USP21 regulates gene transcription by catalyzing the deubiquitination of histone H2A^12^. USP21 also mediates transcriptional initiation of IL-8 by binding to its promoter^13^. RNA virus-induced RIG-1 can be deubiquitinated by USP21^14^. USP21 regulates the stability of transcriptional factor GATA3 and GLI1 through deubiquitination^15,16^.

Mitogen-activated protein kinase kinase 2 (MEK2) is a well-known member of MAPK signaling cascade. Due to the importance of MEK2 in cell proliferation and cell cycle regulation, inhibitors of MEK2 have been applied in several cancer clinical trials^17–19^. However, the effects of USP21 and MEK2 on HCC and the underlying mechanism remain unclear.

In this study, we analyzed the expression of USPs in HCC datasets and found USP21 to be among the most highly expressed relative to its levels in adjacent normal tissues. Furthermore, USP21 was found to interact directly with and deubiquitinate MEK2, and to promote the tumor growth of HCC cells by stabilizing MEK2 and activating ERK1/2 signaling.

## Results

### USP21 is highly expressed in HCCs and associated with poor survival in HCC patients

**Fig. 1:**
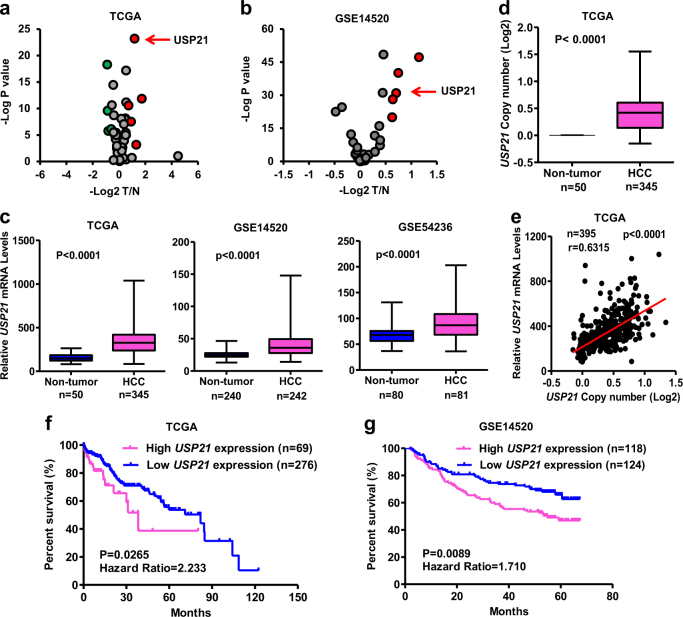
USP21 is amplified and correlates with poor survival in Human HCCs. **a** Volcano plots of 51 USPs relative expression in HCCs, based on TCGA RNA-sequencing data. In TCGA, 5 USPs exhibited significantly differential abundance (*p* < 0.001, absolute fold change > 1.5) when comparing HCC tumors with adjacent normal liver tissues. Red and green indicate high and low expression level in HCC, respectively. **b** Volcano plots of 35 USPs relative expression in HCCs, based on GSE14520 RNA-sequencing data. 5 USPs exhibited significantly differential abundances (*p* *<* 0.001, absolute fold change >1.5), which are indicated with red color. **c** Box plots of USP21 expression in normal liver and tumor tissues in three independent cohorts of HCC cases. **d** Box plots of USP21 gene copy number in normal liver and tumor tissues from TCGA. **e** Correlation between copy number variation and USP21 expression change in tumor compared to non-tumor tissue, based on TCGA data. **f**, **g** Kaplan–Meier Overall Survival curve based on USP21 expression in two independent datasets of HCC, namely TCGA (**f**) and GSE14520 (**g**). Cases were devided into two groups using the minimum *p* value approach. Unless noted, *p* values are based on Student’s two-tailed *t*-test

To search for driver DUBs in HCC, we downloaded several HCC datasets and analyzed USPs expression profiles. In TCGA (containing data of 51 USPs), 5 USPs, including USP49, USP54, USP21, USP35 and USP22, exhibited significantly differential abundance when comparing HCC tumors with adjacent normal liver tissues (Fig. 1a). In GSE14520 (containing data of 35 USPs), 5 USPs, including USP1, USP14, USP21, USP3, and USP11, were highly expressed in HCC tumors (Fig. 1b). USP21 was the only USP that was found to be upregulated in HCCs compared with adjacent liver tissue in both TCGA and GSE14520 datasets (Fig. 1a, b). Increased USP21 expression was further validated in three independent cohorts of HCC cases (Fig. 1c). Expression also tended to be highly correlated with *USP21* gene copy number in HCCs where it was often amplified (Fig. 1d, e). This suggested that dysregulated USP21 expression is the result of gene amplification.

After stratifying all HCC patients from the TCGA and GSE14520 datasets into high USP21 expression group and low USP21 expression groups, Kaplan–Meier analysis indicated that those individuals whose tumors expressed the highest USP21 transcript levels had significantly poorer overall survival (OS) than those with low USP21 (TCGA *P* = 0.0265 and GSE14520 *P* = 0.0089) (Fig. 1f, g). Taken together, our results show that the *USP21* is amplified and overexpressed in a significant fraction of HCCs and correlates with a significantly poorer prognosis.

### Ectopic USP21 expression promotes hepatocyte cell proliferation and tumor growth

**Fig. 2:**
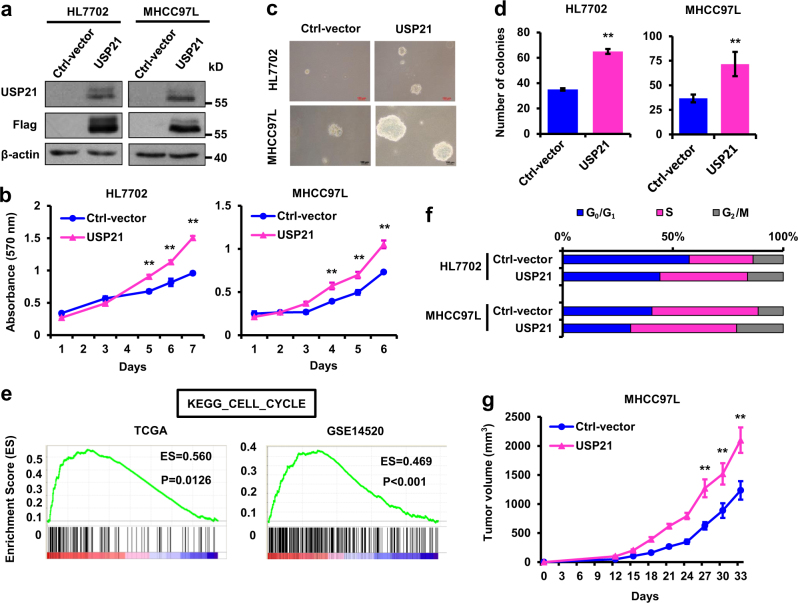
Ectopic USP21 expression promotes cell proliferation and tumor growth. **a** USP21-transduced HL7702 and MHCC97L cells were tested for the presence of FLAG-tag by Western blot. **b** Growth curves of USP21-transduced HL7702 and MHCC97L cells. **c**, **d** Representative images and quantification of anchorage-independent growth of USP21-transduced HLL702 and MHCC97L cells. **e** Gene set enrichment analysis demonstrated that predefined gene sets involved in cell cycle were significantly enriched in HCC cases exhibiting high USP21 expression, suggesting that cell cycle was preserved in these cases. ES, enrichment score. **f** Cell cycle analysis of USP21-transduced HLL702 and MHCC97L cells. Cells were harvested in log phase growth. Nuclei were stained with propidium iodide and analyzed via flow cytometry. **g** Tumor volume growth by 1 × 10^7^ subcutaneous injected USP21-transduced MHCC97L cells. Data in **b**, **d** and **g** are mean, mean ± s.e.m. *n* = 3 mice per group in **g**. Statistical significance was determined by a two-tailed, unpaired Student’s *t*-test. Unless noted, *p*-values are based on Student’s two-tailed *t*-test: **p *<* 0.01

To investigate whether USP21 is functionally involved in HCC cell proliferation, we established normal human hepatocyte HL-7702 and HCC MHCC97L cell lines stably expressing ectopic USP21 (Fig. 2a). As shown in Fig. 2b–d, ectopic USP21 expression markedly increased cell proliferation and anchorage-independent growth.

To better elucidate the function of USP21 in HCC cell proliferation, gene set enrichment analysis (GSEA) was performed. Predefined gene sets involved in cell cycle progression were significantly enriched in HCCs expressing high levels of USP21, as follows: KEGG_CELL_CYCLE in TCGA (*p* = 0.0126) and GSE14520 (*p* < 0.001) datasets (Fig. 2e). Also, USP21 overexpression significantly increased G_0_/G_1_ to S phase progression in HL7702 and FHCC98 cells (Fig. 2f). These results demonstrate that USP21 promotes cell proliferation by inducing cell cycle progression in HL-7702 and MHCC97L cell lines.

We then compared the tumor growth potential of HCC cells. MHCC97L cells, transfected with either empty vector or USP21-FLAG, were subcutaneously injected into nude mice. Ectopic USP21 expression significantly accelerated the rate of tumor growth (Fig. 2g). These results show that the overexpression of USP21 is sufficient to increase HCC cell proliferation in vitro and promote tumor growth in vivo.

### USP21 inhibition decreases HCC cell proliferation and tumor growth

**Fig. 3:**
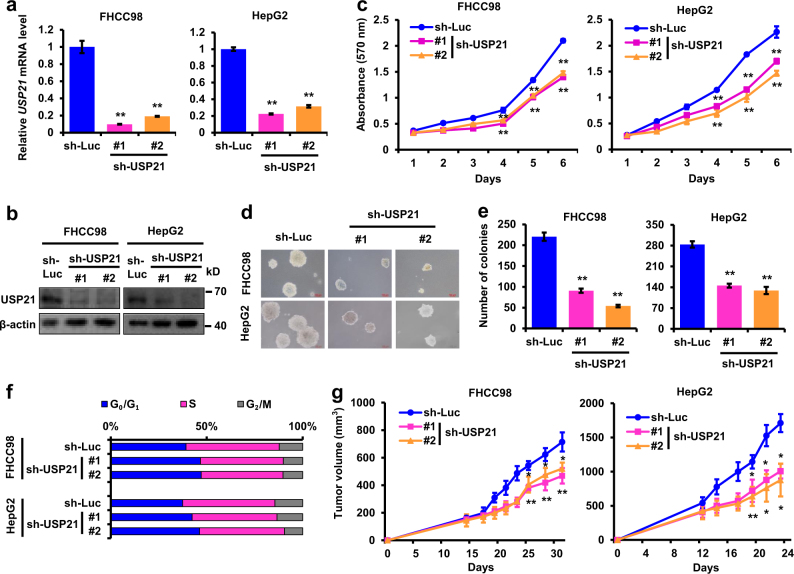
USP21 knockdown suppresses HCC cell proliferation in vitro and in vivo. **a**, **b** Relative expression of USP21 in USP21 shRNA-transduced FHCC98 and HepG2 cells were examined by Real-time PCR and western blot. **c** Growth curves of USP21 shRNA-transduced FHCC98 and HepG2 cells. **d**, **e** Representative images and quantification of anchorage-independent growth of USP21 shRNA-transduced FHCC98 and HepG2 cells. **f** Cell cycle analyses of USP21 shRNA-transduced FHCC98 and HepG2 cells. Cells were harvested in log phase growth. Nuclei were stained with propidium iodide and analyzed via flow cytometry. **g** Tumor volume growth by 2 × 10^6^ subcutaneous injected USP21 shRNA-transduced FHCC98 and HepG2 cells. *n* = 3 mice per group in **f**. Statistical significance was determined by a two-tailed, unpaired Student’s *t*-test. Unless noted, *p*-values are based on Student’s two-tailed *t*-test: *p *<* 0.05, **p *<* 0.01

We further investigated the effects of USP21 modulation on HCC development. Because USP21 is highly expressed in FHCC98 and HepG2 cells, these two cell lines were exploited for further study. shRNA-mediated inhibition of USP21 significantly retarded cell proliferation (Fig. 3a–c), reduced anchorage-independent growth (Fig. 3d, e), increased the accumulation of cells in the G_0_/G_1_ phase (Fig. 3f) in vitro and further inhibited in vivo xenograft growth (Fig. 3g).

### USP21 interacts with MEK2

**Fig. 4:**
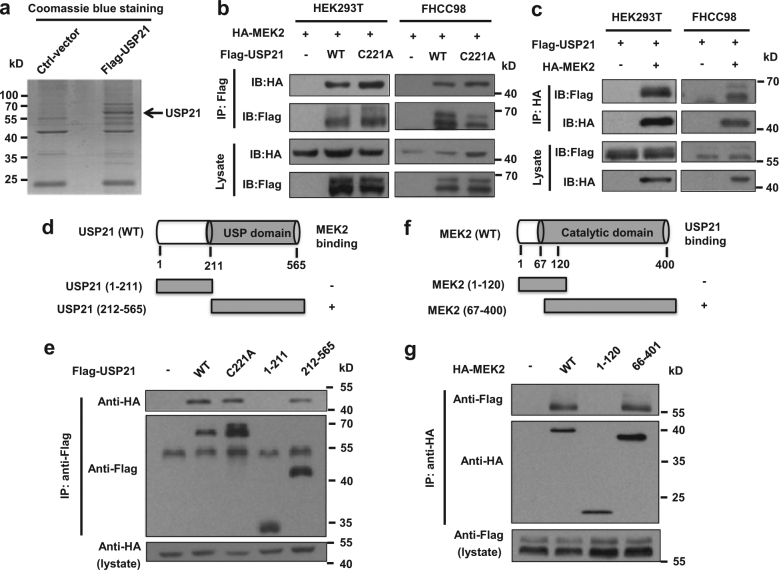
USP21 interacts with MEK2. **a** Identification of USP21-FLAG-associated proteins by mass spectrometry. HEK293T cells were transfected with control vectors or expression vectors encoding FLAG-tagged USP21. USP21-FLAG in the cell lysates was immunoprecipitated with anti-FLAG antibodies and subjected to 10% SDS-PAGE. Coomassie blue-stained USP21-FLAG proteins were identified by Q Exactive HF mass spectrometry. **b** HEK293T and FHCC98 cells were transfected with HA-MEK2 alone or in combination with FLAG-tagged USP21 or USP21^C221A^ mutant, and then immunoprecipitated with FLAG beads and immunoblotted with antibodies against FLAG and HA. **c** HEK293T and FHCC98 cells were transfected with USP21-FLAG alone or in combination with HA-tagged MEK2, and then immunoprecipitated with HA beads and immunoblotted with antibodies against HA and FLAG. **d** Schematic representation of FLAG-tagged full-length USP21 and its various deletion mutants. **e** HEK293T cells were co-transfected with HA-MEK2 and FLAG-tagged USP21 full-length or its deletion mutants, and then immunoprecipitated with FLAG beads and immunoblotted with HA and FLAG antibodies. **f** Schematic representation of HA-tagged full-length MEK2 and its various deletion mutants. **g** HEK293T cells were co-transfected with USP21-FLAG and HA-tagged MEK2 full-length or its deletion mutants, and then immunoprecipitated with HA beads and immunoblotted with antibodies against FLAG and HA

To determine the underlying molecular mechanism of USP21 function in promoting both normal hepatocyte and HCC growth, we immunoprecipitated the FLAG-tagged USP21 from lysates of HEK-293T cells transfected with USP21 and identified the co-immunoprecipitated proteins with USP21-FLAG by mass spectrometry (Fig. 4a). We found that MEK2 was one of the major companion proteins that co-purified with USP21. To validate this funding, co-immunoprecipitation assays were used to detect the interaction between ectopically expressed Flag-tagged USP21 and HA-tagged MEK2. In the former case, we used either wild-type USP21 or a C221A mutation which has been shown to lack de-ubiquitinase activity We confirmed that USP21 and MEK2 could interact reciprocally and in a manner that is independent of its DUB activity (Fig. 4b, c).

To map the binding regions on USP21 and MEK2, we co-expressed HA-tagged MEK2 along with two deletion mutants of USP21, which expressed different functional domains (Fig. 4d). Co-immunoprecipitation assays demonstrated that the C terminal USP domain of USP21 is essential for interaction with MEK2 (Fig. 4e) and that the C-terminal catalytic domain of MEK2 is essential for interaction with USP21 (Fig. 4f, g).

### USP21 deubiquitylates MEK2

**Fig. 5:**
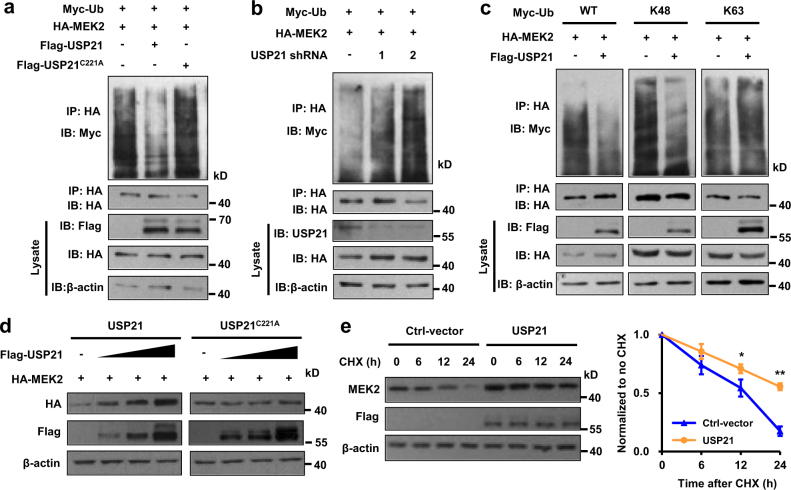
USP21 deubiquitylates and stabilizes MEK2. **a** HEK293T cells were co-transfected with HA-MEK2, Myc-Ubiquitin (Ub) and USP21-FLAG or USP21-FLAG^C221A^ mutant, and then immunoprecipitated with Myc beads and immunoblotted with antibodies against Myc and HA, cells were treated with MG132 (20 µM) for 6 h before collection. **b** HEK293T cells were co-transfected with HA-MEK2, USP21 shRNA and Myc-Ubiquitin (Ub), and then immunoprecipitated with HA beads and immunoblotted with antibodies against Myc and HA. Cells were treated with MG132 (20 µM) for 6 h before collection. **c** HEK293T cells were co-transfected with HA-MEK2, USP21-FLAG and Myc-Ubiquitin Lys 48-only (Ub-K48) or Myc-Ubiquitin Lys 63-only (Ub-K63), and then immunoprecipitated with Myc beads and immunoblotted with antibodies against Myc and HA. Cells were treated with MG132 (10 µM) for 6 h before collection. **d** HEK293T cells were transfected with HA-MEK2 and increasing concentrations of USP21-FLAG. Immune blot was performed with the indicated antibodies. **e** Left: HEK293T cells transfected with the indicated plasmids were treated with cycloheximide (100 µg/mL), and collected at the indicated times for western blot. Quantification of MEK2 levels relative to β-actin is shown. Statistical significance was determined by a two-tailed, unpaired Student’s *t*-test. *p *<* 0.05, **p *<* 0.01. h hour

Since USP21 functions as a deubiquitylase, we next ask whether USP21 deubiquitylates MEK2. HA-MEK2 and Myc-ubiquitin expression vectors were co-transfected with FLAG-USP21 wild type or deubiquitinase-deficient C221A mutant into HEK293T cells. HA-MEK2 proteins were immunoprecipitated with an anti HA epitope antibody and immunoblotted with and anti-Myc-tag antibody to detect the presence of ubiquitinated MEK2. As shown in Fig. 5a, wild type USP21 decreased MEK2 ubiquitination, whereas the C221A USP21 deubiquitinase-deficient mutant failed to do so. Conversely, USP21 inhibition increased MEK2 ubiquitylation in cells (Fig. 5b). Altogether with the findings presented in Fig. 4, these results indicate that at least two distinct regions of USP21 are necessary for it to interact with and deubiquitinate MEK2.

Ubiquitin has seven lysine residues as points of ubiquitination. Of these, K48-linked polyubiquitin chains target proteins for degradation, whereas K63-linked chains are associated with regulatory functions^20^. We next investigated which type of ubiquitin chain of MEK2 was affected by USP21. MEK2 and USP21 expression vectors were co-transfected with ubiquitin wild type or function mutant into HEK293T cells. We found that USP21 efficiently removed Lys 48-linked polyubiquitylation, but not Lys 63-linked polyubiquitylation of MEK2 (Fig. 5c). Taken together, USP21 deubiquitylates Lys 48-linked polyubiquitylation of MEK2.

### USP21 stabilizes MEK2

Since USP21 removed the Lys 48-linked polyubiquitynation of MEK2, we reasoned that USP21 might affect the stability of MEK2. To verify this hypothesis, MEK2 with increasing concentrations of USP21 was introduced into HEK293T cells and the protein levels were detected by immunoblot. As shown in Fig. 5d, the protein levels of MEK2 positively correlated with the increasing concentrations of co-expressed USP21, indicating that USP21 stabilized MEK2 in a dose-dependent manner that is dependent on USP21 DUB activity. In cycloheximide chase experiments, we next showed that the overexpression of USP21 prolonged the half-life of MEK2 (Fig. 5e). Collectively, these and the foregoing findings point to USP21 as being a mediator of MEK2 stability by virtue of its activity as a deubiquitinase.

### USP21 activates ERK1/2 signaling through MEK2

**Fig. 6:**
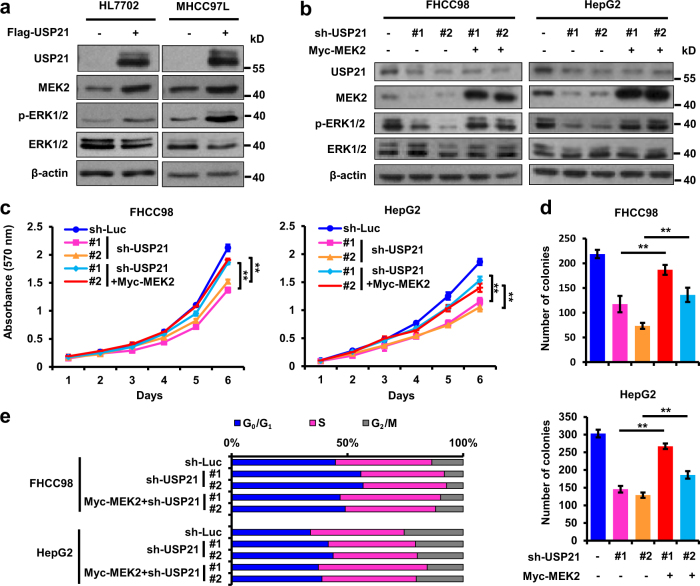
MEK2 mediates USP21 oncogenic phenotypes. **a** Immunoblotting of USP21, MEK2, p-Erk1/2, Erk1/2, p-Erk1/2 and β-actin in HL7702 and MHCC97L cells transfected with retrovirus carrying empty vector or USP21-FLAG. **b** Immunoblotting of USP21, MEK2, p-ERK1/2, ERK1/2 and β-actin in USP21 shRNA-transduced FHCC98 and HepG2 cells with or without ectopic expression of MEK2. **c** Growth curves of cells from **b**. **d** Quantification of soft agar colony of cells from **b**. **e** Cell cycle analyses of cells from **b**. Unless noted, *p*-values are based on Student’s two-tailed *t*-test: **p *<* 0.01

To gain mechanistic insights into USP21 function in HCC, we asked whether USP21 can modulate MEK2 stability in HCC-derived tumor cells. Consistent with our previous findings, USP21 overexpression in hepatoma cell lines resulted in increased MEK2 expression (Fig. 6a). On the other hand, USP21 inhibition resulted in reduced MEK2 expression (Fig. 6b). Overexpression of USP21 also enhanced the phosphorylation levels of ERK1/2, without affecting their total protein levels, which is consistent with MEK2 being the immediate upstream activator kinase of ERK1/2^21^. Interestingly, the down-regulated p-ERK1/2 caused by USP21 inhibition was rescued by MEK2 overexpression (Fig. 6b), thereby suggesting MEK2 to be a direct mediator between USP21 and ERK1/2.

### MEK2 mediates USP21 malignant phenotypes

Since MEK2 can rescue the down-regulation of ERK1/2 phosphorylation induced by USP21 inhibition, we investigated whether USP21 functions as a tumor-promoting protein by regulating MEK2. HCC cell lines were stably transfected with the MEK2 expression vector and the two USP21 shRNAs (Fig. 6b). We observed a pronounced increase in cell proliferation and anchorage-independent growth in USP21 knockdown cell after MEK2 overexpression (Fig. 6c, d). Consistent with this, MEK2 overexpression partially restored cell cycle arrest in USP21 knockdown HCC cells (Fig. 6e) further supporting a role for MEK2 as a mediator of USP21-dependent cell growth. Altogether, these findings strengthen the idea that USP21 promotes cell cycle progression and tumor growth by stabilizing MEK2 and thereby activating ERK1/2 signaling.

## Discussion

In the current work, we have determined that ubiquitin-specific protease USP21 is amplified and upregulated in HCC and that this is inversely correlated with patient survival in two different large patient groups. Previous studies have established that USP21 is an oncogene in some malignancies, including kidney and bladder cancer as well as HCC^13,22–24^. Consistent with our results, the *USP21* gene was also previously reported to be amplified in metastatic urothelial carcinoma^22^.

Here, we have shown that USP21 over-expression promotes HCC cell growth, cell cycle progression and in vivo tumorigenesis (Fig. 2), while knockdown of USP21 inhibits these processes (Fig. 3). In renal carcinoma cells, USP21 has been reported to deubiquitinate microtubule affinity-regulating kinase (MARK) proteins, which further promote the well-known Hippo pathway^23^. On the other hand, USP21 also mediates transcriptional initiation of IL8 leading to an expansion of the stem cell pool in renal carcinoma cells^13^. USP21 has recently been found to deubiquitinate BRCA2, which promotes DNA repair in HCC^24^. Of note is that BRCA2 overexpression was unable to fully restore HCC tumor cell growth following USP21 knockdown. This result indicates that USP21 probably has other targets in HCC. In this study, we identified USP21 as a DUB that specifically regulates its novel substrate MEK2 and activates ERK1/2 in HCC.

The regulation of tumor suppressor and oncoprotein function and stability by differential ubiquitination is a common theme in many cancers. Other deubiquitinases, such as USP16^25^, USP33^26^, and CYLD^27^ were reported to be related to HCC and function as tumor suppressors. Unlike USP33, which has been reported to inhibit mitogen-activated protein kinase (MAPK) activation pathway and suppress hepatoma cell growth^26^, our study suggests that USP21 activates ERK1/2 to promote HCC cell proliferation.

The MEK/ERK signaling pathway regulates multiple cellular processes such as proliferation, survival, differentiation and transformation. These kinases are the last components of a signaling module comprised of RAS, RAF and MEK. Unlike colorectal cancer or melanoma^28,29^, in which mutations in RAS and/or RAF are quite common, these mutations occur in <5% of HCCs^30^. Nonetheless, the MEK/ERK pathway is activated in nearly half of HCC tumor samples^31,32^ thus raising the question as to the basis for this up-regulation. Activation of MAPK signaling pathway has been reported to contribute to the progression of HCC^31^. Regarding clinical therapies for HCC, great importance has been attached to mediators in MAPK pathway, inhibitors of which have been applied in clinical trial^33,34^. Deubiquitinating enzymes, such as USP2A, USP8 and USP15, have been shown to play crucial regulatory roles on maintainence of MAPK pathway through deubiquitination and stabilization of specific effectors^35^.

We now show that USP21 interacts with MEK2, and regulates the Lys48-linked polyubiquitination of MEK2. The resultant stabilization of MEK2 thereby up-regulates ERK1/2 to provide a sustained proliferative and oncogenic signal. Together, these studies provide a mechanistic understanding as to how the non-mutational, post-translational modification of MEK2 lead to the sustained oncogenic signal that is important in some human HCCs.

Other than oncogenic function, USP21 has been reported to participate in different cellular signaling pathways. Stabilization of Nanog by USP21 is crucial for maintaining the “stemness” of murine embryonic stem cells^36^. USP21 prevents the generation of T-helper-1-like Treg cells by deubiquitinating GATA3, which in turn stimulates FOXP3 transcription or directly interacts with FOXP3^15,37^. In the immune system, USP21 negatively regulates antiviral response by deubiquitinating RIG-1^14^. In addition, Hedgehog signaling is regulated by USP21 through deubiquitination of Gli1^16^. Collectively, these functions of USP21 may support a more general role in non-neoplastic proliferation and malignant transformation.

In summary, we have shown that USP21 is highly upregulated in HCCs and is critical for the development of HCC. Mechanistically, USP21 physically associates with MEK2 and stabilizes MEK2 by deubiquitination, thereby contributing to the activation of the MEK2 substrate ERK1/2. Our findings provide a better understanding of HCC progression and identify a novel strategy for the clinical treatment for HCC by targeting the USP21-MEK2 interaction and its functional consequences.

## Materials and methods

### Cell culture and transfection

Human HCC cell lines (MHCC97L, FHCC98, and HepG2) were purchased from The China Center for Type Culture Collection (Wuhan, China) and cultured under standard cell culture conditions in Dulbecco-modified Eagle’s minimum essential medium (D-MEM) (GIBCO, Life Technologies, Grand Island, NY, USA) supplemented with 10% fetal bovine serum (FBS; GIBCO), 1% l-glutamine, 1% penicillin-streptomycin, and 1% nonessential amino acids in a 5% CO_2_-humidified chamber. Human HEK-293T cells were cultured, as described previously^38^. Normal human hepatocytes HL-7702 were grown in RPMI1640 (GIBCO) supplemented with 10% FBS and 1% penicillin-streptomycin, maintained at 37 °C and 5% CO_2_. For transfection, cells were seeded in 6-well plates and transfected with various plasmids by using Lipofectamine 2000 (Invitrogen, Carlsbad, CA,USA), according to the manufacture’s instructions.

### Plasmids and shRNA

Full-length USP21 ORF, USP21C221A and two deletion mutants were subcloned into the pHAGE-CMV-MCS-PGK vector. Full-length MEK2 ORF and two deletion mutants were subcloned into the pcDNA3.1-HA (Invitrogen). The shRNA sequences are listed as follows. sh-USP21 #1: 5′-GCCTTTCTACTCTGATGACAA-3′; #2: 5′-CCACTTTGAGACGTAGCACTT-3′. All constructs were confirmed by DNA sequencing.

### Reagents and antibodies

MG132 (M7449), Cycloheximide (C7698), and Hexadimethrine bromide (H9268) were purchased from Sigma-Aldrich (St. Louis, MO, USA). Mouse monoclonal antibodies against Flag (Sigma-Aldrich, St. Louis, MO, USA), HA (Promotor Biotechnology, Wuhan, China), and β-actin (Santa Cruz Biotechnology, Dallas, TX, USA); rabbit monoclonal antibodies against HA (Sigma-Aldrich); rabbit anti-ERK1/2 and p-ERK1/2 (Cell Signaling Technology, Danvers, USA); rabbit anti-USP21 and rabbit anti-MEK2 (Proteintech Biotechnology, Wuhan, China). Anti-Flag affinity agarose, anti-HA affinity agarose (Selleck, Houston, USA).

### Immunoprecipitation and immunoblot analysis

For immunoprecipitation assays, HEK293T cells and FHCC98 cells transfected with the indicated vectors were lysed in 1 mL RIPA lysis buffer (50 mM Tris–HCl, pH 7.4, 150 mM NaCl, 1 mM EDTA, 5 mM EGTA, 1% Nonidet P-40, 0.25% sodium deoxycholate, 10 μg/mL aprotinin, 10 μg/mL leupeptin, and 1 mM phenylmethylsulfonyl fluoride). For each immunoprecipitation, the indicated gels were washed with 1 mL RIPA Lysis Buffer three times, then 0.9 mL of cell lysate was incubated overnight at 4 °C with constant agitation with 20 μL indicated affinity agarose conjugate. Immunoprecipitated protein was washed three times with lysis buffer and resuspended in 2×SDS sample buffer. The immunoprecipitates and whole-cell lysates were analyzed by immunoblotting with the indicated antibodies.

### Lentivirus infection

Lentiviruses carrying shRNA targeting human USP21 lentiviral vectors (PLKO.1) were from GeneChem (Shanghai, China). Lentiviruses containing overexpressing lentiviral vectors were constructed and packaged using standard procedures. Subsequent lentiviral infections were performed in the presence of Polybrene. After 48 h, cells were cultured in medium containing puromycin for the selection of stable clones. The levels of UPS21 expression in stable clones were then verified by real-time PCR or Western blot.

### Mass spectrometry

After Coomassie Blue staining, excised gel pieces were subjected to in-gel trypsin digestion^39^ and dried. Samples were reconstituted in 5 µl of HPLC solvent A (2.5% acetonitrile and 0.1% formic acid). A nanoscale reverse-phase HPLC capillary column was created by packing 5 µm C18 spherical silica beads into a fused silica capillary (100 µm inner diameter; ∼20 cm length) with a flamedrawn tip. After the column was equilibrated, each sample was loaded onto the column by a Famos autosampler (LC Packings). A gradient was formed and peptides were eluted with increasing concentrations of solvent B (97.5% acetonitrile and 0.1% formic acid). Eluted peptides were subjected to electrospray ionization and then entered an LTQ Velos ion-trap mass spectrometer (Thermo Fisher, Waltham, USA). Peptides were detected, isolated and fragmented to produce a tandem mass spectrum of specific fragment ions for each peptide. Peptide sequences (and hence protein identity) were determined by matching protein databases with the acquired fragmentation pattern by using the software program SEQUEST (Version 28, Thermo Fisher). Mass tolerance was set to 2.0 for precursor ions and 1.0 for fragment ions. The database searched was the Human IPI database (version 3.6). The number of entries in the database was 160,900, which included both the target (forward) and the decoy (reverse) human sequences. Spectral matches were filtered to contain a less than 1% false discovery rate at the peptide level based on the target-decoy method. When peptides matched to multiple proteins, the peptide was assigned so that only the most logical protein was included. The same principle was used for isoforms when present in the database. The longest isoform was reported as the match.

### In vivo ubiquitination assay

HEK293T cells were transiently transfected with HA-MEK2, FLAG-USP21, and the hemagglutinin (Myc)-Ub, Myc-Ub-K48O, or Myc-Ub-K63O expression plasmids. At 18 h posttransfection, the cells were treated with 20 mM MG132. Samples were harvested at 24 h posttransfection and lysed using a RIPA lysis buffer as immunoprecipitation. The samples were denatured by heating for 10 min in 1% SDS and diluted with lysis buffer until the concentration of SDS was decreased to 0.1%. The diluted supernatants were then immunoprecipitated overnight at 4 °C with constant agitation with 20 μL anti-HA-agarose conjugate. Immunoprecipitated protein was washed three times with lysis buffer and resuspended in 2×SDS sample buffer. The immunoprecipitates and whole-cell lysates were analyzed by immunoblotting with various antibodies that are indicated.

### Real-time PCR

Total RNA from transfected HepG2 or FHCC98 cells was isolated using TRIzol reagent (Invitrogen). After reverse transcription with Oligo(dT) primer using a RevertAidTM First Strand cDNA Synthesis (Thermo Scientific, Waltham, USA), aliquots of products were subjected to real-time PCR analysis to measure mRNA expression levels of tested genes. β-actin was used as a reference gene. Gene-specific primer sequences were the listed as following: β-actin- ATCATGAAGTGTGACGTGGACAT (forward) and AGGAGCAATGATCTTGATCTTCA (reverse); USP21- GAGCCTTTCTACTCTGATGAC (forward) and CTCAGACAGAAGTCCCGAAGA (reverse); MEK2-GGTCACCAAAGTCCAGCACAG (forward) and GCTCGCGGATGATCTGGTTC (reverse).

### Soft agar growth, MTT and cell cycle assay

For soft agar colony assays, 3 × 10^3^ cells were planted in triplicate in soft agar (0.35% low melting point agarose on top of 0.7% bottom agarose) in 6-well plates and fed with DMEM. Colonies were counted and photographed after 2 weeks. For MTT assays, 10^3^ cells were planted in triplicate into 96-well plates and grown in DMEM supplemented with 10% FBS The next day medium was carefully replaced on fresh DMEM + 10% FBS with diluted MTT (1:10, 10% MTT), and incubated for 5 h at 37 °C. After removing incubation medium, formazan crystal was dissolved in 200 μl solution of DMSO. MTT reduction was quantified by measuring the light absorbance at 570 nm using the ELx800 absorbance microplate reader. Cell cycle analyses were performed on propidium iodide-stained nuclei using aLSRFortessaX20 (Beckman Coulter, Brea, USA). Data were analyzed by single-histogram statistics^40,41^.

### Xenograft growth

All animal studies were approved by the Animal Care Committee of Wuhan University. Four-week-old male BALB/c nude mice were purchased from SLAC Laboratory Animal (Changsha, China) and maintained in microisolator cages. For xenograft experiments, 2 × 10^6^ or 1 × 10^7^ cells were suspended in 100 µL serum-free DMEM and injected subcutaneously in the flanks of animals (*n* = 3 per group). Tumor growth was monitored every three days for a total period of 20 to 35 days. Tumor volumes were calculated by the equation V (mm^3^) = *a* × *b* × *c*/2, where *a* is the length, *b* is the width, and *c* is the height. Tumors were harvested for DNA, RNA, and protein assays, as well as standard pathological studies^42^.

### Protein half-life assay

For the MEK2 half-life assay, Lipofectamine 2000 transfection was performed when HEK293T cells in 2 cm plates reached about 60% confluence. Plasmid encoding USP21 or empty vector plasmid were used in transfection as indicated in individual experiments. After 24 h, the cells were treated with the protein synthesis inhibitor cycloheximide (Sigma-Aldrich, 100 µg/mL) for the indicated durations before collection.

### Public clinical datasets and gene set enrichment analysis (GSEA)

For USP expression analysis, expression values were downloaded from the public TCGA portal (https://tcga-data.nci.nih.gov/tcga/). Data for 51 USPs were available from a total of 345 HCC tumors. Relative fold change was calculated between primary tumors and non-tumor tissues. For USP21 expression analysis, USP21 mRNA levels were analyzed from TCGA and the National Cancer for Biotechnology Information Gene Expression Omnibus (GEO) database (accession codes GSE14520 and GSE54236). For correlative analysis between USP21 mRNA levels and copy number, data were obtained from the public TCGA database. USP21 clinical data were from TCGA and GSE14520 dataset. Gene set enrichment analysis (GSEA) is a kind of gene enrichment method considering the full list of genes different from single gene method^43^. In GSEA, genes are ranked by their correlation with phenotype and every enrichment gene set will get an enrichment score. GSEA was performed to gain the biological functions and pathways involved in HCC pathogenesis through USP21 pathway. The canonical pathways gene sets (c2.cp.v4.0.symbols.gmt) from the Molecular Signatures Database-MsigDB (http://www.broad.mit.edu/gsea/ msigdb/index.jsp) were used for enrichment analysis.

### Statistics analyses

For data analysis, the SPSS statistical package for windows (SPSS 16, SPSS Incorporated, Chicago, IL, USA) and the GraphPad Software (Version5.01, San Diego, CA, USA) were used. Data are expressed as the mean ± SD or SEM. Other statistical analysis was performed using the two-tailed Student’s *t*-test. *p* *<* 0.05 was considered statistically significant.
